# From Patient Voice to Organizational Adaptability: A Critical Narrative Review and Conceptual Model for Personalized Care

**DOI:** 10.3390/healthcare14183064

**Published:** 2026-09-17

**Authors:** Zakhar Akulov, Zelimkhan Berikkhanov, Maria Sukhanova, Valeria Niskova, Evgeniy Tarabrin, Andrey Nikolaev, Vadim Razumovsky, Alexey Shestakov, Aleksey Kotelnikov, Milena Ivanova, Sara Nourmahal, Sergey Muraviev

**Affiliations:** Department of Hospital Surgery No. 2, Sechenov First Moscow State Medical University (Sechenov University), 119048 Moscow, Russia; berikkhanov_z_g@staff.sechenov.ru (Z.B.); sukhanova_m_a@student.sechenov.ru (M.S.); niskova_v_a@student.sechenov.ru (V.N.); tarabrin_e_a@staff.sechenov.ru (E.T.); nikolaev_a_m@staff.sechenov.ru (A.N.); razumovskiy_v_s@staff.sechenov.ru (V.R.); shestakov_a_l@staff.sechenov.ru (A.S.); kotelnikov_a_g@staff.sechenov.ru (A.K.); ivanova_m_yu_1@staff.sechenov.ru (M.I.); nurmakhal_s@student.sechenov.ru (S.N.); muravev_s_yu@staff.sechenov.ru (S.M.)

**Keywords:** person-centered care, patient voice, patient expectations, current needs, organizational adaptability, care planning, patient-reported information, treatment burden, healthcare management

## Abstract

Hospital routines can elicit patients’ views without providing a reliable route for acting on them. The aim of this article is to develop a positive organizational model in which patient voice provides the basis for tailoring care, while professional responsibility remains with the clinical team and healthcare organization. We conducted a critical narrative review with conceptual synthesis, drawing on a previously assembled corpus of 105 full-text publications and a targeted update completed on 10 August 2026. The analysis compared patient input, organizational responses and limits of transferability. Search documentation was incomplete, and effects were not pooled. The framework distinguishes four types of input, five request dispositions, seven organizational actions and three response domains. These are working constructs informed by literature and conceptual reasoning. The proposed process retains the patient’s wording, assigns responsibility, explains constraints and checks the result. It places recovery, function and relief of suffering at the center of organizational decisions. Implementation could use existing nursing and care-management roles or a dedicated coordinator, with protected time and clear clinical boundaries. We propose patient and professional co-design followed by a prospective multisite feasibility pilot. The pilot would examine response completeness, accessibility, workload and measurement feasibility. A subsequent comparative study would evaluate goal concordance, quality of life, adherence, satisfaction, safety and costs. The accompanying pilot specification provides candidate questions, coding procedures and progression criteria. No pilot data or evidence of the model’s effectiveness are reported.

## 1. Introduction

Personalization has an author. In clinical documents, that author is often the organization. It decides which needs count, when to ask about them and which answers the electronic form will accept. When no open response is allowed, the patient can only choose the nearest option. Even a perfectly completed questionnaire may therefore reflect the limits of its designers’ imagination.

Research has long shown that care should take account of biography, values, relationships, life circumstances and outcomes that matter to the person [1,2,3,4,5]. The difficulty is translating this principle into appointment schedules, delegated authority and available services. Courtesy, information and a choice from a fixed menu all have value. Each can coexist with routines that give the patient’s answer little influence over care [6,7,8].

An administrative perspective makes this system easy to report. It constructs a “typical patient,” averages that patient’s requests and offers a standard response. An actual person brings a different logic. Today, they may need to understand the cause of pain and obtain relief. A few hours later, the pressing questions are whether they can drink, use the toilet, contact family and find out what will happen in the morning. After discharge, returning to work, retaining independence and keeping treatment burden tolerable may come first. None of these concerns has to remain the main priority throughout the care journey.

Dependence on care further distorts the picture. A patient may stay silent to avoid seeming difficult, damaging the relationship with the team or jeopardizing access to treatment. Receiving information does not by itself constitute participation, and the absence of objection does not demonstrate agreement [9,10]. Shared decision-making and coproduction offer a stronger basis, although their effects depend on what happens after the conversation, across departments and at reassessment [11,12,13].

The managerial purpose is to help the person recover. Completing a sequence of procedures is insufficient evidence that care has served that purpose. Recovery may mean regaining function, returning to a valued activity or living with an acceptable treatment burden. When cure is not possible, the same orientation includes comfort, dignity and relief of suffering. Asking what helps or obstructs that process gives everyday concerns a place in clinical and organizational decisions [3,4,5].

Personalization therefore requires patients to be heard in their own terms and organizations to act on what they hear. The team must clarify the request, consider constraints, assign responsibility and review the result. It need not fulfill every wish. It must provide a reasoned response and notice when an earlier answer no longer applies. Whether repeated elicitation improves care, and how often it should occur, are questions for empirical testing.

The aim of this article is to develop a positive organizational model in which patient voice provides the basis for tailoring care, while professional responsibility remains with the clinical team and healthcare organization. The analysis asks who authors the language of personalization, how patient input can influence decisions across services and how the resulting model can be tested without shifting coordination work to patients or families.

## 2. Materials and Methods

### 2.1. Review Design

We conducted a critical narrative review with conceptual synthesis. This design was chosen because the subject spans conceptual work, qualitative studies, observational evidence, trials of complex interventions, implementation research and economic evaluations. The aim was to compare mechanisms and the limits of transferability, not to inventory every publication or calculate a single pooled effect [14,15,16].

The critical element was the comparison of what an approach allows patients to express with what the organization subsequently does. Conceptual synthesis was used to connect these observations into a working model [16]. This is an interpretive review of published literature. It includes no new patient survey, implementation trial or external validation of the model.

### 2.2. Sources and Search Transparency

The starting point was a previously assembled collection of 105 accessible full-text publications concerning patient- and person-centered care, patient voice, shared decision-making, organizational change, recovery and the economics of care. It had been assembled for a related question about organizational decoupling, including gaps between declared patient-centeredness and actual care. It was relevant because accounts of failed implementation also identify conditions that a response process would need to address. Reuse does not provide an independent body of evidence or demonstrate comprehensive coverage.

The targeted update was completed on 10 August 2026. The documented routes were PubMed/MEDLINE records, reference lists and article pages on publishers’ platforms. The cited literature is hosted on platforms including Wiley Online Library, PLOS, Springer Nature, ScienceDirect, JAMA Network, BMJ Journals and SAGE Journals. These article-level access routes should not be interpreted as exhaustive searches of each platform. The update addressed personalized care planning, patient priorities, goal setting, service modularity, implementation of shared decision-making, symptom reporting linked to a clinical response, treatment burden and economic outcomes.

The literature used in this synthesis is English-language. The original search start date, complete database-specific strings and a full record of retrieval, deduplication and exclusions could not be reconstructed from the retained documentation. Appendix A makes the topic combinations explicit through representative PubMed expressions. These were reconstructed for reporting and are not an exact historical search log. The date of the targeted update is therefore a documented endpoint, not evidence that all preceding literature was searched. The 52 references cited in this article are a selected bibliography, including methodological sources, and should not be read as the outcome of screening 105 records.

### 2.3. Relevance and Limits of Selection

Selection was purposive. Sources were relevant if they described how patients express priorities or needs, how this information changes a decision or service, why the transition fails, or which implementation and resource conditions affect it. Eligible forms of evidence included qualitative and mixed-methods studies, observational studies, trials of complex interventions, evidence reviews and conceptual analyses. Methodological papers informed the review design. Reports of neutral or unfavorable findings were retained when they challenged the proposed mechanism.

Clinical studies without a communication or organizational link, and accounts of molecular personalization without a connection to patient voice, were outside the scope. A source had to provide enough information to interpret its contribution and context. There was no uniform minimum sample size or follow-up threshold. Pediatric evidence was used only for explicitly bounded organizational comparisons, and philosophical work was treated as conceptual argument. Incomplete records prevent reconstruction of study-by-study exclusion decisions or claims of independent duplicate screening.

### 2.4. Conceptual Analysis and Evidence Mapping

For each source used to support a component, the analysis considered setting, design, relevant patient information or organizational process, direction of findings and limits of transferability. Empirical observations were separated from the authors’ proposed response rules. Evidence about a related process supports the plausibility of a component, not the effectiveness of the assembled model.

The analytical route combined comparison with theory-informed interpretation. Accounts of desired future outcomes, anticipated care, immediate difficulties and preferred ways of receiving care were compared by function and time horizon. We used these distinctions to define goals, expectations, current needs and delivery preferences. Overlap was retained when one statement could serve more than one function. For example, walking may be an immediate functional need and part of a longer-term goal. This is an illustrative boundary case, not a reported participant quotation.

The five dispositions were specified as organizational rules for making a response traceable. Care-planning and shared decision-making literature informed acceptance and modification by agreement. Reassessment, coordination and autonomy provided the rationale for deferral, referral and explained refusal. No reviewed study established this exact five-part classification. Likewise, the seven actions arrange elicitation, clarification, feasibility assessment, configuration, responsibility and review into a proposed workflow. Maintaining the patient-derived vocabulary is a continuing design function. The actions may overlap or be revisited, and urgent care takes precedence over their sequence.

The three response domains combine person-centered care and service modularity with a normative distinction between essential care, adjustable support and optional amenities. Their boundaries depend on clinical need and setting. Component counts are design choices, not frequencies estimated from the literature. We used interpretive conceptual synthesis rather than formal thematic analysis. No independent coding dataset, reliability calculation or adjudication record is available for this review. Independent coding and examination of unmatched requests are specified for the future pilot in Appendix B.

No protocol was registered. PRISMA was not used because this work is neither a systematic review nor a scoping review. No common formal risk-of-bias instrument was applied, and effects were not pooled. SANRA was used only as an editorial check of the rationale, aims, search description, referencing, argumentation and presentation of evidence [17]. Biases arising from this approach are discussed in Section 4.6.

## 3. Conceptual Synthesis

The synthesis proposes a route from patient expression to an accountable organizational response. Its components are presented below with their literature basis, the limits of that basis and their implications for routine work. The framework remains open to revision, including categories that merge, divide or fail to capture important requests.

### 3.1. Components of the Proposed Model

Patients need a vocabulary that can accommodate what they actually want to say. A question bank can draw on interviews, complaints, suggestions, observation and repeated conversations. At each encounter, a short relevant set of questions should leave room for an unanticipated answer. Table 1 distinguishes four types of input. The categories guide the response and are not intended to classify patients.

Each material request needs a recorded disposition. Accepted means that an action has been agreed. Modified means that a feasible alternative has been agreed with the patient. Deferred requires a reason and a review date or trigger. Referred requires a receiving service to acknowledge responsibility. Declined requires an explanation and, when relevant, a route for review. A disposition records the decision, not proof that the request was fulfilled. Deferred and referred requests remain open until their subsequent outcome is known.

These five dispositions are author-proposed rules. Acceptance and modification draw on negotiated care planning and shared decision-making [11,19]. Deferral makes the need for reassessment explicit [24]. Referral addresses distributed responsibility [25,26,27]. Explained refusal reflects autonomy and the obligation to discuss limits [28,29]. These sources justify the functions, not an exhaustive taxonomy or a tested five-category instrument.

Responses draw on three domains. The protected clinical core includes warranted treatment, safety, dignity and essential support. Configurable support adjusts how care is delivered, such as its timing, communication or coordination. Optional services provide additional amenities that are unnecessary for safe and equitable care. The same service can belong to different domains depending on need. A private room required for infection control, for example, cannot be treated as an optional upgrade. The distinction is an organizational proposal informed by person-centered care and service modularity [3,4,5,6,30].

**Figure 1:**
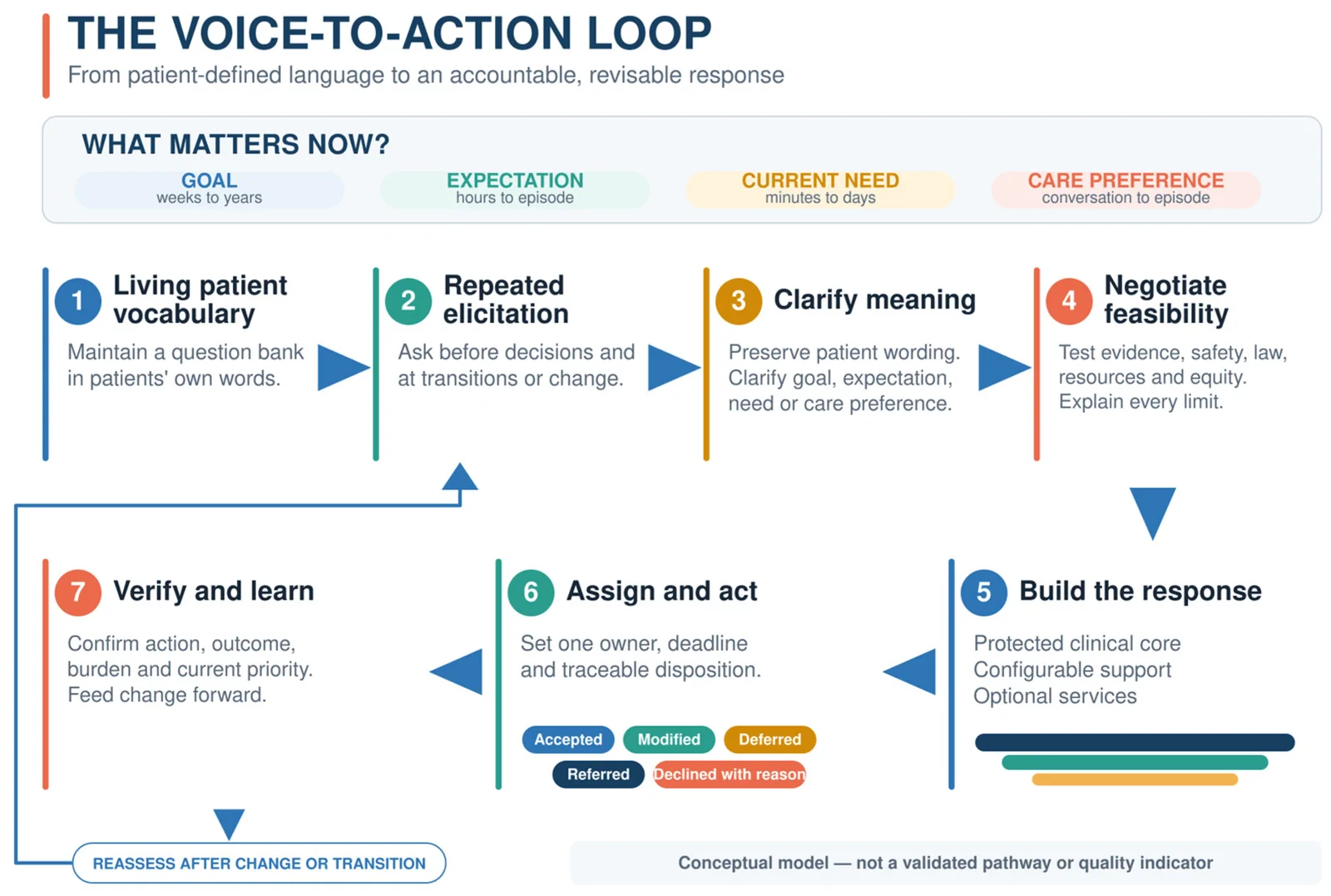
The voice-to-action loop. Seven proposed organizational actions connect patient-defined language to an accountable and revisable response. Vocabulary development is ongoing, and operational actions can overlap or recur. Urgent clinical care takes precedence. The framework has not been validated as a clinical pathway or quality indicator.

Figure 1 connects these components through seven organizational actions. Patient-derived language is maintained, input is elicited and clarified, limits are discussed, a response is configured, responsibility is assigned and the result is reviewed. Table 2 specifies what needs to remain traceable across these actions.

### 3.2. Evidence Supporting the Components and Its Limits

Patient and professional perspectives can organize the same care differently. In a qualitative study of four Dutch teams caring for children with Down syndrome, professionals described discipline-specific provision, while parents emphasized function and overall well-being [31]. This supports retaining patient language when configuring services. It does not establish the proposed categories or show that changing the service description improves outcomes.

Patient experience surveys provide an incomplete response mechanism when organizations do not use the findings to change care. A review of their use in quality improvement found mostly incremental service changes and limited evaluation of their impact [37]. Reviews of feedback from patient-reported outcome measures found more consistent effects on communication and problem recognition than on final health outcomes [38,39]. These findings justify examining the action that follows a report.

The cumulative complexity model relates patient workload to the capacity available to carry it [40]. Qualitative research with primary care physicians also identifies tensions between patient priorities and professional targets in multimorbidity [18]. These sources support attention to the feasibility of a care plan and to work transferred to patients. They do not establish that a coordinator will reduce that work.

Repeated assessment requires a careful rationale. In Auriemma et al.’s review, preferences were often stable. Among 24 studies with extractable stability data, 17 reported stability above 70%, and changes in health were not consistently associated with changes in preferences [24]. The review concerns end-of-life choices. It supports checking whether an earlier preference still applies, but does not establish rapid changes in everyday inpatient needs or the optimal interval for reassessment. Daily check-ins are therefore a candidate implementation option.

A Cochrane review of 19 studies involving 10,856 participants found small, heterogeneous benefits from personalized care planning. More complete cycles, repeated contacts and involvement of the usual clinician were associated with larger effects, chiefly in chronic disease and primary care [19]. These comparisons support the plausibility of a connected process, but do not isolate which component causes improvement.

Patient Priorities Care provides both supportive and limiting findings. A non-randomized study of 366 older adults associated priorities-aligned care with lower treatment burden and less unwanted healthcare [20]. A later non-randomized controlled study of 264 patients did not demonstrate statistically significant differences in treatment burden, shared prescribing decisions or nonhealthy days [21]. The mixed findings require process changes and patient outcomes to be evaluated separately.

Goal-setting interventions in rehabilitation have not consistently included all components of active patient participation [22]. Service modularity offers a way to configure a package around an individual, but accounts of implementation remain sparse and much of the literature is conceptual [30]. Neither body of evidence establishes a universal package of configurable support.

In the PRO-TECT cluster-randomized trial, 1191 patients at 52 oncology practices reported symptoms, with concerning reports generating care-team alerts. Emergency department use and deterioration in physical function, symptoms and quality of life favored monitoring, while overall survival did not differ [23]. This is evidence for that intervention in oncology. It illustrates a signal-response-review process without establishing an equivalent effect for heterogeneous hospital requests.

Implementation of shared decision-making also depends on agreed roles and integration into routine work. The MAGIC study examined 54 interviews with 31 professionals in three secondary care teams, without an independent patient assessment [25]. Its findings inform workflow design, while the empirical effects of the proposed framework remain unknown. Table 3 summarizes the principal contextual evidence.

### 3.3. Preserving Meaning Across the Response

A short chain should be recoverable for each material request. The record needs the patient’s original wording, its interpretation, the decision, the responsible person, the agreed time and the subsequent outcome. Table 2 maps these requirements to the seven actions. A structured field should not replace the original wording or create another full questionnaire at every contact.

Patients may be unable or unwilling to answer. Pain, fatigue, fear, communication difficulty or a wish to defer discussion can make an apparently simple form inaccessible [9,10,28,29]. Silence does not identify a diagnosis, consent or absence of need. The proposed response is another time, channel or interlocutor, with interpretation or a trusted person when wanted. Declining participation remains a legitimate choice.

Priorities also cross organizational boundaries. At transfer or discharge, the patient’s wording, decision and authority to act may be held by different teams. The receiving team should acknowledge unresolved requests and check whether they remain relevant. This is the proposed continuity function of the model. It prevents a referral from being counted as a completed response merely because it left the sending team’s record.

### 3.4. Organizational Responsibilities

The model needs authority and staff time as well as a way to record requests. Leaders must specify what staff can adjust, which requests require escalation and who can allocate resources. Standards protect safety and consistency, while authorized options allow adaptation within those limits [7,8,26,27,33]. An instruction to individualize care without workable authority or capacity leaves the clinician responsible for a problem the organization has not solved.

Nurses already assess symptoms, identify changes, educate patients, coordinate care and follow up responses. These functions belong within existing clinical and nursing workflows [2,3]. A coordination function should support this work by following requests across services. It should not replace nursing assessment or add an unresourced parallel reporting system. Clinical decisions remain with appropriately qualified professionals.

Two staffing arrangements are proposed in Table 4. In a small department, an existing senior nurse or care manager could coordinate requests during protected time, assisted by trained support staff. A larger center could assign a dedicated response coordinator or service manager. In both arrangements, clinical uncertainty is escalated promptly, and urgent symptoms bypass the routine request queue. A volunteer may assist with communication or recording under supervision but should not carry clinical triage or sole responsibility for continuity.

Training should cover accessible questioning, clarification, recording, routing and explanation of limits. Staff must recognize situations requiring immediate clinical review. Appendix B proposes a 12-h introductory program based on these functions, followed by supervised practice. This is a training plan to test, not an established qualification or staffing standard. Request volume, staff time and missed care should determine whether tasks can be redistributed or extra staffing is required.

## 4. Discussion

### 4.1. Organizing Care Around Recovery

The proposed change is in what the organization regards as a completed piece of work. A procedure may have been delivered correctly while the patient remains unable to understand the plan, tolerate its burden or prepare for life after discharge. Clinical quality remains essential. The additional managerial question is whether the way care is organized helps this person recover, function or obtain relief.

Recovery-oriented questions can make this link visible. For example, patients can be asked which aspects of food, rest, communication or support are helping or hindering recovery, and what change would matter now. An open response should precede any list of suggested services. The list can then help someone articulate an overlooked concern, with space to say that a proposed option is irrelevant or unwanted.

A candidate question bank could cover clinical communication and symptom support, the conditions of care, relationships with staff and additional help the patient would welcome. Table A1 gives examples for testing. These content areas are distinct from the four input categories in Table 1. Questions should allow helpful, unhelpful, no-effect, uncertain and not-applicable responses. Perceived contribution to recovery must be analyzed separately from clinical recovery and satisfaction.

The healthy professional’s perspective can miss the work that illness imposes on someone dependent on care. Education, confidence and freedom to insist should not become hidden eligibility criteria for being heard [1,9,10,28,29]. A useful thought experiment for managers is whether they would accept the same rule while in pain, afraid and dependent. If they would seek an exception for themselves, its clinical basis and availability to comparable patients deserve examination.

The question also applies to familiar routines. Contact with family, access to online information, food preferences or contact with an animal may matter to an individual. The team needs to assess the request, discuss safety and offer a feasible response. The model aims to make these conversations routine, including an explanation when accommodation is impossible.

A large question bank should support brief encounters. Adaptive item banks show how a small relevant set can be selected from a larger pool [42]. This is a technical analogy, not validation of a needs questionnaire. Patients must influence which wording and options enter the bank [32,43]. Repeated contact should follow clinical changes and transitions, with the frequency tested against its burden. The process must remain open to stable priorities as well as changing ones.

### 4.2. Adaptation When Resources Are Limited

The minimum version could use one open question, a paper record, a named recipient and a documented return to the patient. The team would identify the most pressing issue, assess feasible action, give a response within an agreed interval and revisit unresolved requests at handover. Urgent needs require immediate clinical assessment. Routine acknowledgement on the same day could be tested as a local target, but is not an evidence-based universal standard.

Limited capacity does not make every request indivisible. If a private room is unavailable, the team can clarify whether the person mainly needs privacy, quiet or family contact and discuss an acceptable alternative. Partial accommodation needs the patient’s agreement and should be recorded as such. The relevant priorities will differ between elective surgery, chronic care, rehabilitation and palliative care. Local inquiry should determine them rather than assigning a standard profile to each department.

A short check-in could ask about pain, distress, understanding of the plan and anything making recovery or daily functioning harder. It can be incorporated into an existing encounter when this does not compromise clinical work. The useful frequency, duration and number of questions need testing. A paper process avoids dependence on new information technology but still uses staff time. It cannot create a missing interpreter, medicine, transport service or safe staffing level. Such shortages require resource decisions and a visible record of unmet need.

Clinical and managerial experience reported by S.M. in district hospitals and federal centers motivates a hypothesis of ‘personalization inversion’. Familiarity with patients and their families may sometimes help a small institution respond personally despite limited technology, while complex procedures may impede responsiveness in a larger center. This is an experiential observation, not a systematically collected comparison or evidence that rural hospitals perform better. Familiarity can also compromise privacy or disadvantage newcomers. Testing should examine these competing possibilities across settings.

For rural hospitals and lower-resource health systems, the first assessment should cover staffing and continuity, language and literacy, travel and referral constraints and the services to which a request can actually be routed. The same review is needed in well-funded hospitals with fragmented authority. Local patient and staff involvement is necessary because organizational boundaries and available options differ. Implementation should strengthen existing relationships while making responsibility, constraints and unmet needs explicit.

### 4.3. The Patient’s Information Pathway

Patients may bring information from the internet or generative artificial intelligence into the care relationship. Studies of internet-informed patients and assessments of digital answers help explain their appeal [44,45,46]. They do not show that an algorithm understands a particular patient better than the clinical team. Use may reflect curiosity, convenience or a need for explanation, so it should not automatically be interpreted as distrust.

The team can ask what the patient has read, what they found convincing and which advice they intend to follow. Murray et al. found that relationship difficulties were associated with clinicians’ perceived resistance to patients’ internet information [44]. Discussing that information provides an opportunity to examine evidence, uncertainty and conflicts with the care plan. Evaluations of artificial intelligence responses have identified clinically important errors and potentially harmful recommendations [47]. Relevant advice should therefore be discussed again when the patient’s condition or plan changes.

A future request database could identify recurring organizational failures and supply candidate topics for the question bank. Predictive models would be a later extension, evaluated against simple rules and direct questioning. Records from the same patient must remain in one analysis partition, and testing should include a different site or later cohort. Evaluation would examine patient-confirmed relevance, calibration, false alerts and performance across groups. A prediction should invite a conversation, never substitute for an expressed preference. Secondary use would require appropriate authorization and restricted access, with care available without algorithmic profiling.

### 4.4. Proposed Pilot and Comparative Evaluation

**Figure 2:**
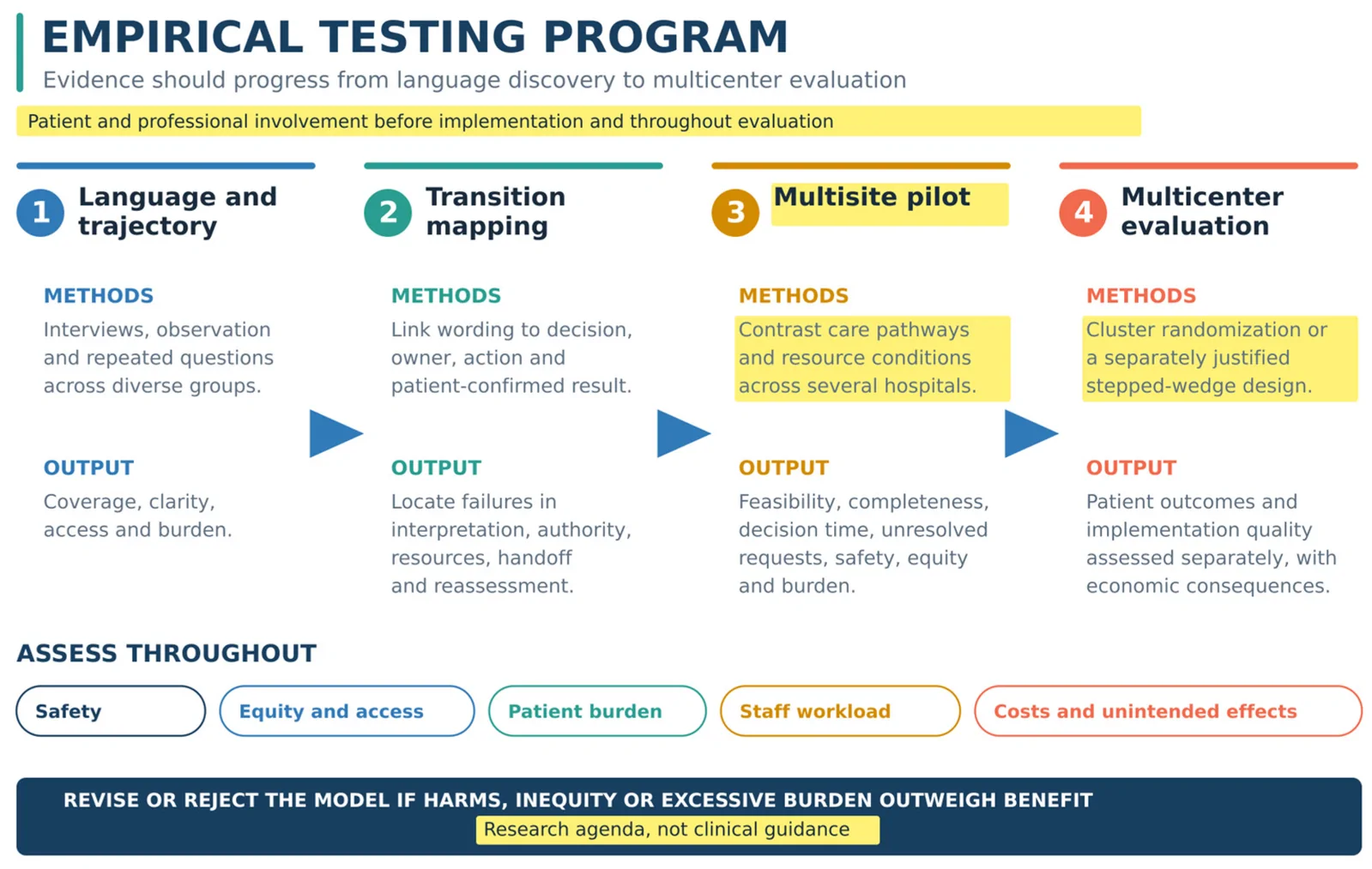
Proposed empirical testing program. Patient and professional involvement precedes implementation and continues through language studies, transition mapping, a multisite feasibility pilot and multicenter comparison. Appendix B specifies the pilot. Site numbers depend on the study objectives and design. Safety, equity, burden and costs are assessed throughout.

We propose a prospective, mixed-methods feasibility study before testing effectiveness. Figure 2 outlines four stages, from development of patient-derived language to multicenter comparison. Appendix B specifies a candidate pilot workflow, questions, coding procedures and progression criteria. Recruitment, stakeholder validation and pilot data collection have not begun.

Patients and caregivers should first work with nurses, physicians, care coordinators and managers to examine relevance, wording and feasibility [3,32]. Participants should include people with limited literacy, disability, language barriers and experience of poorly resourced care. Cognitive interviews would test how questions are understood. Workflow walk-throughs would test routing, handover and clinical escalation. Two independent coders would then examine whether the proposed categories accommodate patients’ statements, with a third reviewer resolving disagreements after initial agreement has been recorded.

The multisite pilot would recruit consecutive eligible adult inpatients from contrasting district and referral settings. The ward or care pathway would be the implementation unit. Participating sites would document usual practice, test local adaptations and then evaluate a stable version of the response process. Site selection would reflect differences in staffing, services and information systems. Neither the number of hospitals nor the total sample is fixed in advance of those feasibility requirements. A precision-based planning example is provided in Appendix B.

The primary feasibility outcome would be participant-level response completeness for one initial priority selected by the patient. A complete record would retain its wording, the agreed interpretation, a disposition, a responsible person, a review time and the subsequent result checked with the patient. Explained refusal may complete a response process, but cannot be counted as fulfillment. Missing patient confirmation would remain visible. Other requests would be retained as secondary records so that choosing an index priority does not restrict care.

Elicitation would occur once the patient is clinically ready, with a brief daily check-in tested within an existing encounter and reassessment after meaningful changes. The team would review unresolved requests at handover and discharge. Candidate patient outcomes would be collected at common prespecified times, including a proposed 30-day follow-up. Table 5 distinguishes feasibility, patient outcomes and balancing measures. The core response pathway and any coordination provided through the pilot would be available without patient charges.

The pilot would assess recruitment, implementation, data completeness and burden, not establish clinical effectiveness. Results would be reported by site and study phase. Repeated requests from one person are not independent observations. Baseline and implementation comparisons would describe workflow change, with secular trends, case mix and measurement effects acknowledged. Each adaptation cycle would record the intended change, observation and decision to retain or revise it [50].

Table 6 translates six development proposals into questions that can be tested separately. The initial pilot concentrates on elicitation, a traceable response and coordination. Physiological monitoring, predictive algorithms and financing arrangements require later studies. Heart-rate variability, skin conductance and salivary cortisol would need independent validation against an appropriate assessment of distress. Neither these measures nor handwriting features are established indicators of an individual’s need for personalization in the reviewed evidence.

A definitive study would follow only after the response process and outcome assessment are workable. Cluster randomization could limit contamination between staff caring for different patients. A stepped-wedge design would need a separate rationale and control for time trends. Both groups would receive the same independent outcome assessment at the same times. Goal-concordance measures require particular care because published definitions and methods differ [34,35,36]. The 3D trial illustrates why improved care processes do not necessarily improve quality of life [48]. One primary patient outcome, a clinically meaningful target difference, follow-up, missing-data rules and a cluster-adjusted sample size should be specified before allocation.

The program is intended to support collaborative testing across institutions. Common definitions would permit comparison while preserving a record of local adaptations. Progression would depend on usable responses, acceptable burden and safe, equitable access, with provisional progression criteria specified in Table A3. An unfavorable result would prompt redesign or rejection of the relevant component.

### 4.5. Economic Consequences and Optional Services

Patient experience and financial performance are distinct outcomes. Richter and Muhlestein found an observational association between experience and hospital profitability [41]. Reviews also link experience with safety and effectiveness, but satisfaction has substantial measurement limitations and does not ensure favorable utilization or survival [49,51,52]. These findings support an economic research question, not a claim that personalization pays for itself.

Economic evaluation should compare implementation and training costs, coordination time, healthcare use and work performed by patients and families. Staff time has an opportunity cost even when no new post or software is purchased. Length of stay, medication use, complications and complaints can be measured, but their reduction cannot be assumed. Costs and outcomes should first be reported separately, with uncertainty and a clearly stated perspective. Local estimates are needed before calculating budget impact or return on investment.

Optional amenities may generate revenue where permitted, but their full delivery cost and effect on access must be measured. Essential clinical coordination, interpretation, disability support, symptom relief and clinically necessary psychological care belong within the protected service. An optional personal service manager can assist with additional nonclinical arrangements only if all patients retain a functioning route for essential requests. Ability to pay must not determine whether a clinical concern is heard, escalated or followed up.

The proposed separation of essential support and optional amenities should itself be evaluated. Relevant measures include fulfillment of essential requests in both paying and nonpaying groups, staff time diverted from core care, net revenue after costs and any difference in waiting or access. Resources released or generated could support training and coordination, but this remains a conditional financing option. The reviewed literature supplies no defensible universal prices, savings, payback period or staffing ratio.

### 4.6. Limitations

This review depends on purposive selection and author interpretation. Reusing a corpus assembled around organizational decoupling may favor accounts of failure and make a connected response model appear more necessary than a differently selected literature would suggest. Full-text availability and backward citation searching can reinforce familiar authors, institutions and conceptual traditions. The targeted update broadens the discussion but cannot quantify or eliminate these biases. Claims that a new search would recover the same evidence are unwarranted.

Publication bias may favor successful interventions and well-resourced institutions that can publish implementation work. Unpublished failures, routine adaptations and unfavorable economic consequences may be missing. Including the neutral findings of the later Patient Priorities Care study [21] limits a one-sided interpretation but does not correct publication bias. No registry-based or grey-literature audit was performed, and no quantitative assessment of this bias is possible here.

Language bias also limits transfer. Reliance on English-language literature may omit locally developed approaches and culturally different accounts of autonomy, family involvement and institutional authority. The absence of non-English evidence from this bibliography is not evidence that relevant work does not exist. Applicability to Russia, rural institutions and lower-resource countries therefore requires direct study and a broader multilingual evidence search.

The evidence is uneven across settings and designs. Chronic disease and primary care dominate the care-planning literature, rehabilitation informs goal setting and serious illness informs much of the work on preference stability and concordance. Oncology illustrates a responsive symptom-monitoring intervention. Philosophical analyses inform the ethical rationale. These forms of support cannot be treated as equivalent evidence for a whole-hospital intervention, and no common formal appraisal or pooled estimate was produced.

The four input types, five dispositions, seven actions and three domains remain working constructs. External validation has not been completed. Patients may identify topics outside the model or find some distinctions unhelpful. Appendix B is a proposed feasibility specification, including illustrative training cases and provisional progression thresholds. It supplies no evidence that the questionnaire, staffing arrangements or model are effective. Experiential observations about rural practice likewise cannot establish comparative performance.

Several findings would count against the model. Patient-generated wording might add no useful information to existing instruments. Repeat questions could increase burden without changing decisions. A coordinator could accelerate administrative closure while leaving priorities unmet. Recording may narrow conversation, algorithms may reproduce majority preferences and paid services may worsen inequality. A complete economic assessment could show that the additional work is unjustified. These are reasons to revise or reject components, not effects to exclude from evaluation.

## 5. Conclusions

Personalization should help the person recover, retain function or obtain relief. It begins when the patient can describe what matters now and the organization can show how that information affected a decision. Meaning must be clarified, constraints explained, responsibility assigned and the outcome revisited. The proposed framework gives these tasks a traceable form while preserving clinical accountability.

A standard clinical core can coexist with feasible adjustments to care. An explained refusal or an unchanged plan may be appropriate, but a recorded disposition alone does not demonstrate that the person was heard or helped. Nurses, clinicians and coordinators need defined responsibilities and sufficient time. Patients and families should not become the default managers of fragmented services.

This critical narrative review proposes a model for collaborative development and empirical testing. Patient and professional validation should precede a multisite pilot across contrasting settings, followed by comparative assessment of meaningful outcomes, safety, equity, burden and cost. A more explicit account of recovery gives that evaluation its purpose. Whether the proposed organization of care achieves it remains to be established.

## Figures and Tables

**Table 1:** Four types of patient input, their literature basis and proposed use.

Patient Input	Time and Elicitation	Literature Basis and Limitation	Proposed Organizational Use
Life or health goal	Weeks to years. Elicit when choosing a strategy and after a meaningful change.	Priority alignment and rehabilitation goal setting address desired outcomes [18,19,20,21,22]. They do not validate this category across all settings.	Link the plan to a desired future, such as returning to work or retaining independence.
Expectation of treatment or stay	Hours to an episode. Elicit before an intervention, transfer or discharge.	Communication and participation research addresses information needs and barriers to asking [4,9,10]. Separating expectations from preferences is our interpretation.	Clarify what the patient expects to happen and explain likely events, uncertainty and feasible options.
Current need	Minutes to days. Ask at admission and after a clinical or functional change.	Physical and emotional support are established care dimensions [3,4]. Symptom monitoring illustrates a timely response [23]. Everyday requests are broader.	Assess and route pain, nutrition, toileting, mobility, distress or a current barrier to care.
Preference for how care is delivered	One conversation to an episode. Revisit when the context changes.	Person-centered frameworks and shared decision-making address individual preferences and relationships [1,2,3,4,5,11]. The proposed boundary remains flexible.	Adapt timing, information, communication channel and involvement of a trusted person.

Time horizons are illustrative. The sources inform the distinctions, but none validates this exact typology. The examples are explanatory, not participant quotations. Categories can overlap or change during care.

**Table 2:** Proposed response chain with its literature basis and points of failure.

Proposed Action	Related Literature	Minimum Traceable Record	Potential Failure
1. Maintain patient vocabulary	Patient involvement and coproduction [12,13,31,32]	Original wording, source of a new item and reason for revising the question bank	Minority or unexpected answers cannot change the vocabulary.
2. Elicit and revisit	Care planning, stability of preferences and symptom reports [19,23,24]	Time, channel, current priority and whether the patient wishes to participate	Silence or an old answer is treated as continuing agreement.
3. Clarify meaning	Information, autonomy and participation [4,9,10,11,28,29]	The request in the patient’s words, its intended meaning and any necessary support	A staff category displaces the meaning or an accessibility barrier is missed.
4. Discuss feasibility	Implementation barriers and autonomy [7,8,29]	Clinical or resource constraint, explanation and the person authorized to decide	A hidden rule or uneven access determines the response.
5. Configure a response	Care planning and modular services [19,30,31]	Relevant clinical core, adjustable support and any agreed alternative	The available package remains unrelated to the stated need.
6. Assign and act	Implementation and organizational roles [25,26,27,33]	Owner, deadline, disposition and acknowledgement by any receiving service	Referral or a completed field is counted as fulfillment.
7. Reassess and learn	Repeated contact and concordance measurement [19,23,24,34,35,36]	Action, result, patient assessment, burden and whether the priority still applies	Closure is recorded without checking outcome or changed circumstances.

The ordering is a design proposition, not an observed or validated sequence. References support related functions. An unchanged plan can be appropriate when it accords with the patient’s priority or follows an explained constraint. Administrative closure, patient agreement and fulfillment must be recorded separately.

**Table 3:** Selected supporting publications and limits of inference.

Evidence Source	Study Type and Setting	Contribution to the Model	Main Limitation
Personalized care planning [19]	Cochrane review of 19 studies involving 10,856 adults	More complete cycles and repeated contacts were associated with larger effects	Mainly chronic conditions and primary care. Small and heterogeneous effects.
Priority alignment, 2019 [20]	Non-randomized study of 366 older adults	Associations with lower treatment burden and less unwanted healthcare	Non-random allocation, limited settings and residual confounding.
Priority alignment, 2024 [21]	Non-randomized controlled study of 264 patients	No significant difference in treatment burden, shared prescribing decisions or nonhealthy days	One intervention and one usual-care site. Small sample and pandemic disruption.
Goal setting [22]	Systematic review of rehabilitation interventions	Active participation involves several coordinated components	Rehabilitation context. Many interventions incompletely supported participation.
Service modularity [30]	Theory building supported by a scoping review	Rationale for configuring care from service components	Few implementation studies. Effectiveness of the proposed configuration is unproven.
Professional and family perspectives [31]	Qualitative study in four Dutch teams for children with Down syndrome	Different organization of discipline-specific provision and family priorities	Pediatric and proxy perspectives. No effectiveness trial.
Electronic symptom monitoring [23]	Cluster-randomized oncology trial in 52 practices with 1191 patients	Improved secondary outcomes with symptom alerts and clinical follow-up	No overall survival benefit. Results concern this oncology intervention.
Shared decision-making implementation [25]	54 interviews with 31 professionals in three secondary care teams	Agreed roles and a shared purpose supported integration into routine work	No independent patient assessment and few teams.
Workload and capacity [40]	Conceptual narrative model	Rationale for assessing the work a care plan imposes on patients	A conceptual account, not a test of the proposed coordinator.
Experience and profitability [41]	Observational panel of 19,792 observations from 3767 hospitals	Rationale for a separate economic evaluation	Association cannot establish financial benefit or exclude reverse causation.

These publications provide empirical examples or conceptual arguments for individual components. They do not validate the complete model. Estimates from different settings are not pooled or transferred to general hospital care.

**Table 4:** Proposed distribution of responsibilities in two organizational settings.

Function	Small Department or District Hospital	Larger Center with a Coordinator	Boundary and Accountability
Elicit and record	Nurse during an existing encounter. Trained support staff may assist.	Nurse or coordinator, using the same shared record.	Anyone identifying urgent symptoms promptly contacts the clinical team.
Assess and clarify	Nurse assesses nursing needs. Physician reviews clinical decisions. Senior nurse or care manager clarifies service requests.	Clinical team assesses clinical needs. Coordinator clarifies nonclinical requests.	A nonclinical coordinator does not diagnose, prescribe or replace nursing assessment.
Decide feasibility	Responsible clinician and department lead decide within their authority.	Responsible clinician and service manager or administrator.	Managers authorize resources. Clinical safety remains with qualified staff.
Coordinate action	Named senior nurse or care manager with protected time and cover.	Dedicated coordinator follows requests across services.	Receiving services acknowledge ownership. The coordinator tracks progress.
Explain and reassess	Clinical team explains clinical decisions. Named coordinator returns service responses.	Clinical team and coordinator retain the same division.	Patient feedback, unresolved requests and changes are included at handover.
Train and review	Department lead reviews workload, missed care and recurrent obstacles.	Clinical and administrative leads review performance across services.	Training, authority and staffing are adjusted from observed workload and competence.

These are staffing options for local testing, not validated staffing ratios. Assignment depends on competence, workload, local professional scope and available cover. One shared record and named responsibility at handover should prevent duplicate work.

**Table 5:** Candidate measures for pilot and comparative evaluation.

Domain	Candidate Measure and Denominator	Timing	Interpretation
Reach and access	Eligible patients offered accessible elicitation/all eligible patients. Record participation, decline and inability to respond separately.	Admission and transitions	Report by language, disability, setting and other relevant barriers.
Response completeness	Participants whose initial priority has every required response field and patient confirmation/all participants who identified an initial priority.	First review point, no later than discharge	Primary pilot outcome. Missing confirmation is incomplete, not presumed success.
Timeliness and fulfillment	Time to acknowledgement, decision and action. Report fully met, partly met and unmet priorities separately, including clinically appropriate refusals.	Agreed review time and discharge	Keep original deadlines. Referral, deferral or refusal is not fulfillment.
Goal concordance	Patient assessment of whether the decision addressed the recorded priority, paired with independent record review [34,35,36].	After the decision and at follow-up	Report agreement, disagreement and missing assessments. Requires measurement testing.
Quality of life and recovery	EQ-5D-5L as a candidate quality-of-life measure [48], with relevant symptom or function measures selected for the population [42].	Baseline and day 30	Use the specified scoring method. Perceived help is a separate outcome.
Adherence and treatment burden	Agreed care tasks completed/tasks due, summarized per patient. Treatment Burden Questionnaire for participants with an ongoing treatment regimen [20,21].	Baseline and day 30, as applicable	Separate informed refusal, inability, changed plans and missing reports.
Experience and satisfaction	A locally appropriate patient-experience instrument [37,49]. Test a separate 0–10 item on satisfaction with the response to the stated priority.	At discharge, with the same mode in both groups	Report the score distribution and nonresponse. The new item is not a validated scale.
Safety and equity	Adverse events, un-escalated urgent concerns and delays in essential care. Use eligible-patient or request denominators, specified for each indicator.	Continuous monitoring	Review incidents and differences in access, including ability to pay.
Implementation and workload	Minutes by staff role, patient and family time, duplicate documentation, missed care and reasons for non-use.	Baseline, adaptation and stable delivery	Include follow-up work, not only the time spent asking questions.
Resource use and cost	Length of stay, unplanned use, implementation costs and coordination costs per patient. Net revenue from optional services separately.	Episode and day 30, with later economic follow-up if justified	Describe case mix, cost perspective and clustering. No savings are assumed.

This is a measurement menu, not a requirement to administer every instrument to every patient. Response completeness is the primary pilot outcome. The brief recovery and satisfaction questions require testing. Use authorized, population-appropriate language versions of established instruments, report missing data and keep clinical outcomes separate from process measures.

**Table 6:** Comparative questions for staged development.

Question	Intervention and Comparator	What to Measure	Development Stage
1. Earlier elicitation	Admission questions linked to a response versus the existing timing and use of feedback.	Unmet priorities at discharge, response time and burden of questioning.	Core pilot, then controlled comparison
2. Needs-based support	Allocation by current urgency, barriers and expressed need versus the existing allocation process.	Access to requested support, unmet need and staff time across groups.	Feasibility first
3. Physiological information	Optional monitoring plus assessment of distress versus the same assessment alone.	Incremental detection, false alerts, confounding and patient burden.	Separate later study
4. Dedicated coordination	A coordinator versus protected coordination time within current roles, with equal access for patients.	Response time, patient-confirmed outcomes and time spent by each role.	Pilot staffing options, then comparison
5. Predicted questions	Algorithm-suggested prompts versus simple rules and direct questioning.	Patient-confirmed relevance, missed needs, false alerts and external performance.	Only after an adequate governed dataset
6. Optional-service financing	A clearly separated amenity service versus the existing financing arrangement.	Net revenue, core-care access, waiting and diversion of staff time.	Separate economic and equity study

Comparators must describe care actually delivered, including existing nursing coordination and shared decision-making. The questions are not predictions of benefit. The initial pilot does not test all six additions simultaneously, and essential care must never depend on payment, profiling or research participation.

## Data Availability

No new data were created or analyzed in this study. Data sharing is not applicable to this article.

## Appendix A. Reconstructed Search Framework

The expressions below make the topic combinations transparent. They are representative PubMed title-and-abstract queries reconstructed during revision, not recovered queries from the original search. No retrieval counts or selection flow are attributed to them. English-language source use and the documented update endpoint of 10 August 2026 are described in Section 2.2. An original lower date boundary cannot be verified.

***Patient input and care planning***
(“patient-centered care”[Title/Abstract] OR “person-centred care”[Title/Abstract] OR “patient voice”[Title/Abstract]) AND (“care planning”[Title/Abstract] OR “patient priorities”[Title/Abstract] OR “goal setting”[Title/Abstract] OR “shared decision making”[Title/Abstract])
***Feedback and organizational action***
(“patient-reported outcome measures”[Title/Abstract] OR “patient experience”[Title/Abstract] OR “patient feedback”[Title/Abstract]) AND (“quality improvement”[Title/Abstract] OR “organizational change”[Title/Abstract] OR implementation[Title/Abstract] OR response[Title/Abstract])
***Workload and capacity***
(“treatment burden”[Title/Abstract] OR “patient workload”[Title/Abstract] OR “patient capacity”[Title/Abstract]) AND (multimorbidity[Title/Abstract] OR “chronic disease”[Title/Abstract] OR “care coordination”[Title/Abstract])
***Service configuration and economic consequences***
(“service modularity”[Title/Abstract] AND (healthcare[Title/Abstract] OR care[Title/Abstract])) OR ((“patient experience”[Title/Abstract] OR “patient satisfaction”[Title/Abstract]) AND (cost[Title/Abstract] OR profitability[Title/Abstract] OR “resource use”[Title/Abstract]))

## Appendix B. Proposed Feasibility Pilot

This specification is a prospective research proposal for local co-design and protocol approval. It contains no observations, recruited sample or estimated intervention effect. The initial study would test whether an accessible, accountable response process can be delivered without displacing essential care. It would also establish whether the candidate patient outcomes can be collected reliably enough for a later trial.

### Appendix B.1. Sites, Participants and Preparation

Participating wards would include contrasting district and referral settings, with variation in staffing and available services recorded explicitly. Consecutive patients aged 18 years or older would be approached once clinically stable and able to participate through an accessible communication route. Acute instability would delay an approach. Disability, language difficulty or low literacy would prompt support, not automatic exclusion. Where appropriate and locally authorized, a proxy route would be available, with proxy and patient reports kept distinct. Screening would record all eligible patients, reasons for nonparticipation and short stays that prevent a review.

Patient and professional partners would review the questions, routing rules and burden before recruitment. The protocol would obtain required ethics and institutional approvals, specify consent or another approved basis for each data use and protect the right to decline without consequences for care. The ward would name a clinical lead, coordination lead, cover arrangements and an incident reviewer independent of day-to-day delivery. Research records would use coded identifiers, restricted access and a specified retention period.

### Appendix B.2. Delivery and Candidate Questions

The site would first observe usual workflow, then conduct documented adaptation cycles and finally assess a stable version. These phases would be reported separately. The first conversation would ask what matters most today, what is making recovery or daily functioning harder and what the patient would like the team to do. The patient would choose one priority for the primary feasibility record. Table A1 supplies optional prompts, not a fixed questionnaire to administer in full. The proposed large item bank would develop from patient wording, with no requirement to reach a predetermined number of questions.

**Table A1:** Recovery-focused prompts for cognitive testing.

Content Area	Example Wording to Test	Response and Use
Clinical communication and symptoms	“Is anything about your symptoms or the treatment plan making recovery or daily functioning harder?”	Open answer. Clinical concerns go to the clinical team.
Conditions of care	“How are the food, noise or privacy here affecting your recovery or daily functioning, if at all?”	Ask about one relevant factor at a time, then clarify a feasible change.
Relationships with staff	“Is the way staff communicate with you helping, hindering or making no difference to your ability to take part in care?”	Allow uncertainty and a private answer to someone outside the direct care team.
Additional help	“Would you like any additional information or support? What would you want it to help you do?”	Yes, no, unsure or not now. Do not imply that accepting a service improves recovery.

These are proposed examples, not quotations from participants or a validated instrument. Ask an open question first. Where a directional response is useful, allow helping, no effect, hindering, uncertain and not applicable. Ask about one factor at a time and retain the original wording.

At admission, staff would agree who will respond and when. A daily check-in within an existing encounter would ask whether the priority remains relevant, what has happened and whether anything new matters. Patients could defer or decline. Urgent concerns would bypass the research process. At the agreed review time and at discharge, staff would distinguish action taken, patient assessment, partial fulfillment, unmet need and any continuing referral. Day-30 contact would test follow-up feasibility and collect the selected outcomes. An assessor outside the direct response team would obtain the discharge satisfaction item where feasible.

The candidate satisfaction item is ‘Overall, how satisfied are you with the way the team responded to what mattered to you?’ Responses would range from 0, not at all satisfied, to 10, completely satisfied, with ‘unable to judge’ recorded separately. This item measures satisfaction with the response, not recovery or goal concordance. The separate recovery prompts would initially be analyzed item by item. A total score, internal-consistency claim or latent scale should not be assumed for a bank of heterogeneous requests.

### Appendix B.3. Coding and Assessment of Agreement

Structured content coding would test category clarity, while open analysis would examine unexpected concerns. The coding unit would be a meaning-bearing request at a specified time, not a patient type. One statement could receive several input or response-domain codes. Its current disposition would have one recorded value, with later changes retained as a history. Table A2 illustrates decisions that coders and staff would need to distinguish.

**Table A2:** Hypothetical cases for training and coding discussions.

Illustrative Request	Input Type	Possible Disposition and Domain	What Must Be Checked
An explanation of the treatment plan with a relative present	Expectation and delivery preference	Accepted. Clinical core and configurable support.	Patient agrees to the relative’s involvement. The clinician explains the plan and checks understanding.
A private room when none is available	Delivery preference	Modified by agreement. Configurable support.	Clarify whether the need is privacy, rest or family contact. Check whether the agreed alternative actually helped.
A longer planning discussion while the patient is exhausted	Current need and expectation	Deferred. Configurable support.	Agree another time, retain the original request and respond sooner if the situation changes.
Help to manage stairs at home after discharge	Goal and current functional need	Referred. Clinical core.	The relevant clinical or rehabilitation service acknowledges responsibility. Referral alone is not completion.
A preferred food that is currently clinically unsafe	Delivery preference	Declined with explanation. Clinical core.	A qualified clinician explains the specific restriction, discusses safe options and sets a review trigger where relevant.

The cases are constructed examples, not patient records or prescribing recommendations. Dispositions depend on the actual clinical assessment and the patient’s agreement where an alternative is offered. An amenity may be optional when chosen for convenience, but the same provision belongs to essential care when clinically required.

Two trained researchers would independently code all index-priority records in the stable evaluation cohort using a dated codebook. Training examples would be excluded from the agreement analysis. Input and domain labels would be assessed separately as present or absent. The single current disposition would be assessed as a nominal category. Report raw agreement, category frequencies and Cohen’s kappa with uncertainty where estimable, retaining the two original ratings before third-reviewer adjudication. A high aggregate agreement value would not establish validity. Rare categories, unmatched statements and disagreements would guide revisions with patient partners. Agreement for a changed codebook would be assessed on new records.

### Appendix B.4. Feasibility Endpoint, Sample Planning and Analysis

For the primary outcome, the denominator would include every enrolled participant who personally identified an initial priority, including those using communication support. The numerator would require the original wording, agreed interpretation, disposition, owner, review point and patient-confirmed account of the subsequent result. Missing confirmation, including early discharge or later inability to respond, would count as incomplete in the primary estimate, with reasons reported. Proxy-only priorities would form a separate descriptive cohort. Participants who state that they have no request would also be reported separately. This endpoint measures completion of the response process, not fulfillment or clinical benefit.

A proposed precision target is a 95% confidence interval with a half-width of about 10 percentage points for response completeness within each site. For illustration, 100 independent participant records give a Wilson interval of approximately 40.4–59.6% when 50 of 100 are complete. This calculation is a planning benchmark, not an expected completion rate or a fixed total sample. The final target would allow for within-ward dependence, the fraction of patients identifying a priority and local recruitment capacity. More requests from the same patient cannot substitute for more independent participants.

Analysis would report recruitment, completeness, fulfillment and burden by site and phase, with confidence intervals where the design permits. Request-level analyses would account for clustering within patients. Staff time would include recording, handover and follow-up. Deaths, withdrawals, inability to respond and lost contact would be distinguished. Available-case summaries would accompany, not replace, the primary missing-as-incomplete estimate. Exploratory clinical changes would be descriptive. The pilot would not estimate the definitive trial’s target benefit from a small, unstable treatment-effect estimate.

### Appendix B.5. Training and Progression Decisions

The proposed introductory program allocates four hours to accessible questioning and clarification, two to recording and check-ins, four to routing and handover and two to ethics and professional boundaries. Supervised scenarios would test recognition of urgent concerns, explanation of a refusal and transfer of an unresolved request. Twelve hours is a proposed starting allocation, not evidence of competence. Supervision and protected time would continue according to observed workload and performance.

**Table A3:** Provisional criteria for progression beyond the pilot.

Criterion	Proceed Candidate	Amend and Retest	Pause or Redesign
Response completeness	At least 80% in the stable delivery cohort.	60% to below 80%, with correctable causes.	Below 60%, or recurring failures without an accountable owner.
Day-30 assessment	At least 80% of surviving enrolled participants are assessed.	60% to below 80%, requiring changes to contact or measurement.	Below 60%, making the planned endpoint impractical.
Workload	Delivery fits the locally agreed capacity, with no unresolved evidence of missed care.	Excess time or duplicate work has a feasible remedy.	Sustained overload or diversion from essential care.
Category usefulness	Definitions guide action and unmatched requests remain visible.	Recurring ambiguity or missing concepts requires revised definitions.	Classification repeatedly conceals or distorts important needs.
Safety and equitable access	No unresolved safety or access concern after review.	A remediable access barrier requires correction before expansion.	Potentially related serious harm or denial of essential care triggers immediate review.

The numerical thresholds are proposed planning rules, not validated standards or expected findings. Patient and professional partners must approve them before recruitment. Decisions use site-specific estimates, confidence intervals and reasons for failure. A favorable percentage cannot override a safety concern.

An independent review would consider the complete pattern of evidence. Falling below a threshold would lead to a documented explanation and, where feasible, another adaptation cycle. Potentially related serious harm, unsafe delay or denial of essential care would trigger immediate review without waiting for recruitment to finish. A later comparative trial would proceed only after unresolved safety and access problems had been addressed and the intervention and assessment procedures were stable.
